# The evolution and function of the *PSEUDO RESPONSE REGULATOR* gene family in the plant circadian clock

**DOI:** 10.1590/1678-4685-GMB-2022-0137

**Published:** 2022-09-16

**Authors:** Carlos Takeshi Hotta

**Affiliations:** 1 Universidade de São Paulo, Instituto de Química, Departamento de Bioquímica, São Paulo, SP, Brazil.

**Keywords:** Circadian clock, circadian rhythms, pseudo-response regulators, core oscillator, gene evolution

## Abstract

*PSEUDO-RESPONSE PROTEINS* (*PRRs*) are a gene family vital for the generation of rhythms by the circadian clock. Plants have circadian clocks, or circadian oscillators, to adapt to a rhythmic environment. The circadian clock system can be divided into three parts: the core oscillator, the input pathways, and the output pathways. The PRRs have a role in all three parts. These nuclear proteins have an N-terminal pseudo receiver domain and a C-terminal CONSTANS, CONSTANS-LIKE, and TOC1 (CCT) domain. The PRRs can be identified from green algae to monocots, ranging from one to >5 genes per species. *Arabidopsis thaliana*, for example, has five genes: *PRR9*, *PRR*7, *PRR5*, *PRR3* and *TOC1*/*PRR1*. The *PRR* genes can be divided into three clades using protein homology: TOC1/PRR1, PRR7/3, and PRR9/5 expanded independently in eudicots and monocots. The PRRs can make protein complexes and bind to DNA, and the wide variety of protein-protein interactions are essential for the multiple roles in the circadian clock. In this review, the history of PRR research is briefly recapitulated, and the diversity of PRR genes in green and recent works about their role in the circadian clock are discussed.

## Introduction

Plants have an internal timekeeping mechanism that allows them to anticipate periodical events, such as dawn and dusk, track seasons’ passage, and modulate internal and external signals (Farré and Liu, 2013; McClung, 2021). This timekeeping mechanism is called the circadian clock or circadian oscillator. The circadian clock system is usually divided into input pathways, core oscillator, and output pathways. The core oscillator is a regulatory network that generates sustainable rhythms at the cellular level. Even though the core oscillator can run under constant environmental conditions, it can be continually regulated or reset by the input pathways to stay synchronised with environmental rhythms (Webb et al., 2019). Plants with internal rhythms that are not synchronised with external rhythms are less productive and have lower fitness (Dodd et al., 2005). Input pathways bring external cues to the core oscillator, such as light and temperature, or internal, such as sugar levels. The output pathways take the temporal information generated between the core oscillator and input pathways to the rest of the plant.

The core oscillator generates rhythms through a series of interlocked transcriptional-translational feedback loops. The main components of the plant core oscillator are the LATE ELONGATED HYPOCOTYL/ CIRCADIAN CLOCK ASSOCIATED 1 (LHY/CCA1), GIGANTEA (GI), the EVENING COMPLEX (EC), composed of LUX ARRHYTHMO (LUX), EARLY FLOWERING 3 (ELF3) and ELF4, and the PSEUDO-RESPONSE REGULATOR (PRR) family. The PRR gene family comprises five genes in *Arabidopsis thaliana* (L.) Heynh (Brassicales): *AtPRR1*, also known as *TIME OF CAB EXPRESSION 1* (*AtTOC1*), *AtPRR3*, *AtPRR5*, *AtPRR7* and *AtPRR9*. These nuclear proteins have an N-terminal pseudo receiver domain (PR) and a C-terminal CONSTANS, CONSTANS-LIKE, and TOC1 (CCT) domain. The PR domain is similar to the receiver domain of a two-component response regulator, but they lack the characteristic phospho-accepting aspartate site in the receiver domain. However, the PR domain is still necessary for the PRRs to make homo- and heterodimers. The CCT domain is found in 45 Arabidopsis proteins, shares similarities with some histones motifs, and can bind DNA and proteins (Wenkel et al., 2006; Tiwari et al., 2010). The Arabidopsis proteins AtPRR9, AtPRR7 and AtPRR5 also have a motif involved in transcriptional repression in the intermediate region (IR) between their PR and CCT domains (Nakamichi et al., 2010; Wang et al., 2013). The PRRs are essential for the proper function of the plant circadian clock, but the details of their function are still unknown. These genes are frequently targets for selection during breeding, changing the plant perception of the photoperiod (Turner et al., 2005; Beales et al., 2007; Murphy et al., 2011). Here, the early history of PRR research in Arabidopsis, the evolution of this gene family in green plants, and our current understanding of their function in the circadian clock are reviewed.

## Early PRR research in Arabidopsis

The first core oscillator mutant in plants was described in 1995 (Millar et al., 1995). The short-period *toc1*-1, identified in a mutant screening looking for *Arabidopsis* with defects in the luminescence rhythms generated by LUCIFERASE expression under the control of a *CHLOROPHYLL A/B BINDING PROTEIN 2* (*AtCAB2*) promoter (Millar *et al.*, 1995). In 2000, *AtTOC1* was cloned and identified as a PRR, and the *toc1*-1 phenotype resulted from a point mutation in the CCT domain (Strayer et al., 2000). Four other *PRR*s were identified and associated with the core oscillator (Strayer *et al.*, 2000). Later, the *PRRs* were shown to have transcription rhythms during the daytime, with peaks 2 h to 3 h apart, forming “waves of expression”: *AtPRR9* is the first to peak near dawn, then *AtPRR7*, *AtPRR5*, *AtPRR3* and *AtTOC1*, near dusk (Matsushika et al., 2000). In 2001, the first model of a plant core oscillator was proposed as a feedback loop between AtTOC1 and AtLHY/CCA1 (Alabadí et al., 2001). In this early model, AtLHY/CCA1 repressed *AtTOC1* by binding to its promoter, while AtTOC1 would activate *AtLHY/CCA1* expression. At that moment, no DNA binding motif was known in AtTOC1. In 2003, ZEITLUPE (AtZTL) was shown to interact with AtTOC1, targeting it for degradation and changing the core oscillator’s period, the first description of protein-level regulation of the core oscillator (Más et al., 2003). In 2005, AtPRR7 and AtPRR9 were suggested to form an additional feedback loop with AtLHY/CCA1 (Farré et al., 2005).

In 2007, AtPRR3 was found to be expressed only in the vasculature, forming protein-protein complexes with AtTOC1 in competition with AtZTL (Para et al., 2007). In 2009, CCA1 HIKING EXPEDITION (AtCHE) was shown to interact with AtTOC1 while binding to the *AtCCA1* promoter. Thus, AtCHE was suggested to be the molecular link between AtTOC1 and AtCCA1 (Pruneda-Paz et al., 2009). However, AtCHE does not bind to the *AtLHY* promoter, leaving the model incomplete.

In 2010, AtPRR9, AtPRR7, and AtPRR5 were shown to be transcriptional repressors of *AtLHY/CCA1*, despite lacking a typical DNA binding domain (Nakamichi et al., 2010). In these proteins, but not AtTOC1, the IR contained a motif essential for repressing *AtLHY/CCA1* expression (Nakamichi *et al.*, 2010). In the same year, the CCT domain of CONSTANS (AtCO), which was thought as a protein-protein interaction domain, was also shown to bind to DNA (Tiwari et al., 2010). In 2012, AtTOC1 was also described as a transcription factor, acting mainly as a transcriptional repressor (Gendron et al., 2012; Huang et al., 2012). 

In 2013, sugars from photosynthesis were shown to regulate the circadian oscillator through PRR7, in a process called “metabolic dawn” (Haydon et al., 2013). Later, this regulation was shown to be mediated by the transcription factor bZIP63, trehalose-6-phosphate metabolism, and SnRK1/KIN10 (Frank et al., 2018). 

## The evolution of PRRs in plants

**Figure 1 -:**
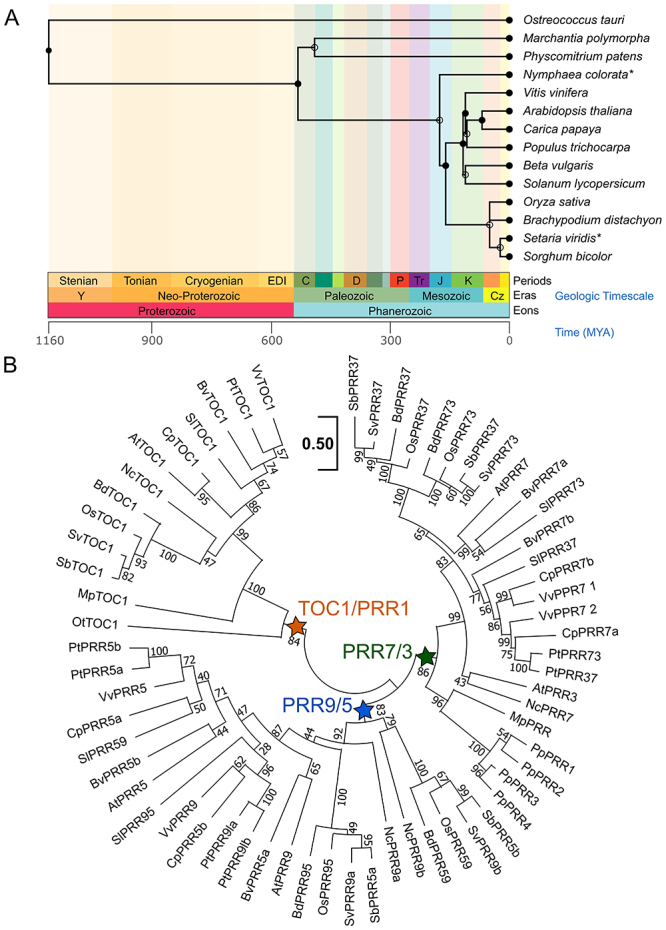
Phylogenetic relations of PRR proteins. **(A)** Timetree of the fourteen species used for sequence analysis (Kumar et al., 2017). * species that were substituted by the species of the same genera. Some branches were flipped for visualisation purposes. **(B)** The phylogenetic tree was built using Maximum Likelihood Bootstrap (500 replicates) after sixty-three PRR proteins from fourteen species were aligned using MUSCLE (MEGA11). Evolutionary distances were calculated using the JTT+F matrix-scale bar, 0.2 substitutions per site. Values at the nodes represent bootstrap support values. The nodes that define the TOC1/PRR1 (orange), PRR7/3 (green) and PRR9/5 clades (blue) are shown as stars. Sequences ID can be found in Table S1.

*PRRs* can be found in all green plants (Viridiplantae) (Table 1). This review analysed the protein sequence of PRR genes from fourteen species to show how this gene family expanded within the green plants (Figure 1). The *PRRs* can be divided into three clades based on their identity: *TOC1/PRR1*, *PRR7/3*, *PRR9/5* (Figure 1B) (Murakami et al., 2003; Takata et al., 2010; Satbhai et al., 2011; Farré and Liu, 2013; Linde et al., 2017).

**Table 1-:** Number of PRR members of each clade in fourteen different species. Numbers in parenthesis correspond to pseudogenes that have sequences similarities. The complete sequence list can be found in Table S1.

Species	TOC1/PRR1 clade	PRR7 clade	PRR9 clade	Total
*Ostreococcus tauri*	1	0	0	1
*Marchantia polymorpha*	1	1	0	2
*Physcomitrium patens*	0	4	0	4
*Nymphaea colorata*	1	1	2	4
*Arabidopsis thaliana*	1	2	2	5
*Carica papaya*	1	2	2	5
*Populus trichocarpa*	1	2	4	7
*Vitis vinifera*	1 (2)	2	2	7
*Solanum lycopersicum*	1	2 (1)	2	6
*Beta vulgaris*	1	2	2	5
*Oryza sativa*	1	2	2	5
*Brachypodium distachyon*	1	2	2	5
*Setaria viridis*	1	2	2	5
*Sorghum bicolor*	1	2	2	5

In green algae, such as *Ostreoccocus tauri* C.Courties & M.-J.Chrétiennot-Dinet, 1995 (Chlorophyta), only one PRR can be found. These algae are believed to have a simple core oscillator: a TOC1/PRR1 ortholog forming a simple feedback loop with an LHY/CCA1 ortholog (Corellou et al., 2009; Thommen et al., 2010). Bryophytes have genes from the *TOC1/PRR1* and the *PRR7/3* clades. *Marchantia polymorpha* L. (liverwort, Marchantiales) has one gene from the *TOC1/PRR1* clade (*MpTOC1*) and one from the *PRR7/3* clade (*MpPRR*). Some circadian oscillator genes have expanded in bryophytes, but some were also lost (Linde et al., 2017). In *Physcomitrium pattens* (Hedw.) Mitt. (synonym: *Physcomitrella patens*, Funariales), four genes from the *PRR7/3* clade (*PpPRR1*, *PpPRR2*, *PpPRR3*, *PpPRR4*) resulted from a recent expansion, but no *TOC1/PRR1* ortholog was found (Holm et al., 2010; Satbhai et al., 2011). The absence of a *TOC1/PRR1* gene is uncommon among vascular plants, but other non-vascular plants share the same loss: *Anthoceros agrestis* Paton (Anthocerotales), *Sphagnum fallax* H. Klinggr. (Sphagnales), *Ceratodon purpureus* (Hedw.) Brid. (Dicranales). It remains to be established how the loss of an essential gene in other species would have on the circadian clock of these species and how this could be compensated. For example, in *M. polymorpha*, loss of the *LHY/CCA1* ortholog is compensated by *DE-ETIOLATED1* (*MpDET1*), arrhythmic in Arabidopsis (Lagercrantz et al., 2021).

**Figure 2-:**
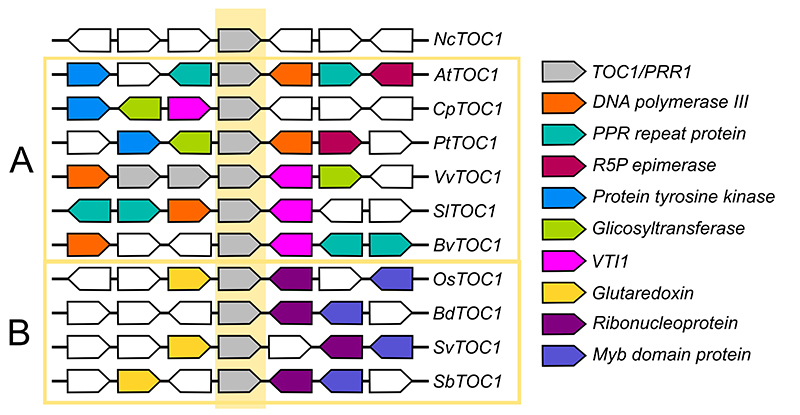
Positional orthology of members of the TOC1/PRR1 clade. The flanking genes of the *TOC1*/*PRR1* orthologs (grey polygons in the yellow centre) of eleven vascular plant clades were identified and colour-coded according to their identity-the polygons point toward the annotated direction of the gene. Two groups of orthologs can be identified through similarities: one for eudicots **(A)** and one for monocots **(B)**. Sequences ID can be found in Table S1.

**Figure 3-:**
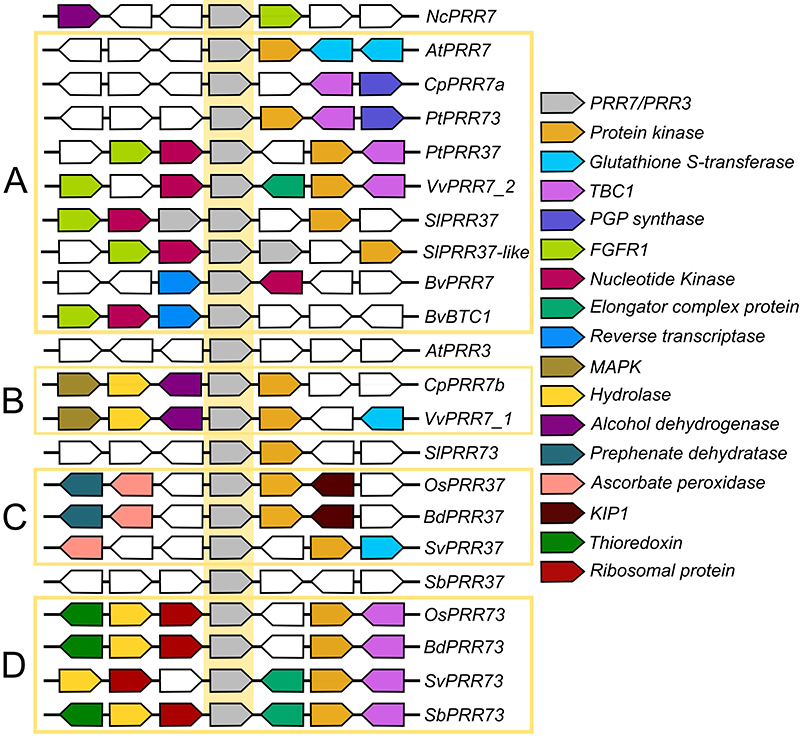
Positional orthology of members of the PRR7/3 clade. The flanking genes of the *PRR7*/*3* orthologs (grey polygons in the yellow centre) of eleven vascular plant clades were identified and colour-coded according to their identity-the polygons point toward the annotated direction of the gene. Four groups of orthologs can be identified through similarities: two for eudicots **(A and B)** and two for monocots **(C and D)**. Sequences ID can be found in Table S1.

**Figure 4 -:**
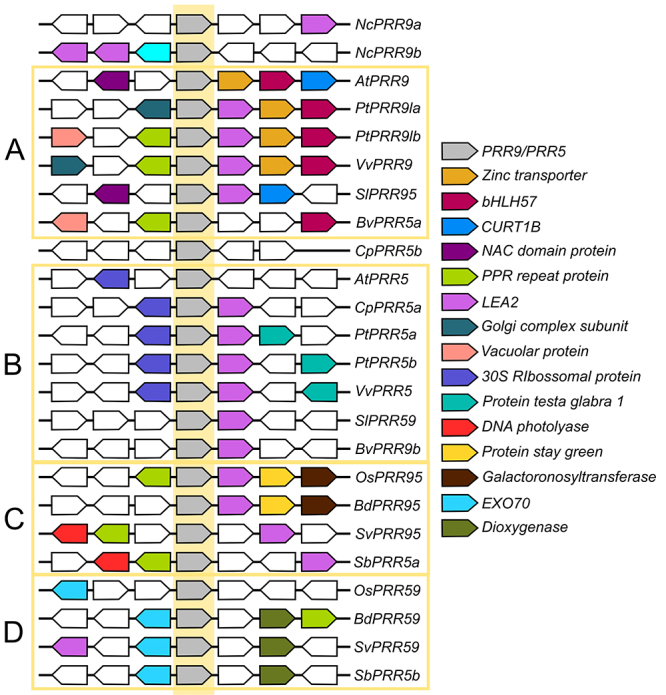
Positional orthology of members of the PRR9/5 clade. The flanking genes of the *PRR9/5* orthologs (grey polygons in the yellow centre) of eleven vascular plant clades were identified and colour-coded according to their identity-the polygons point toward the annotated direction of the gene. Four groups of orthologs can be identified through similarities: two for eudicots **(A and B)** and two for monocots **(C and D)**. Sequences ID can be found in Table S1.

The *PRR9/5* clade only appears in Angiosperms, which usually have one gene from the *TOC1/PRR1* clade (Figure 2) and 2 or 3 genes of the *PRR7/3* (Figure 3) and *PRR9/5* (Figure 4). While the appearance of the PRR7/3 and PRR9/5 clades precedes the Eudicot-Monocot split, their expansion probably happened independently in both groups. Analysis of the eudicot *PRR7/3* and *PRR9/5* gene expansions using chromosomal synteny suggests that it is the result of the γ (gamma) polyploidy event, a whole-genome duplication (WGD) event that occurred early in eudicot divergence (Tang et al., 2008; Takata et al., 2010; Chanderbali et al., 2022). The same analysis suggests that the expansion of the PRR7/3 clade in monocots resulted from the ρ (rho) polyploidy event, but the PRR5/9 clade was duplicated before (Takata *et al.*, 2010). However, the *Nymphaea colorata* L. (water lily, Nymphaeales) genome has only one *PRR7/3* but two *PRR9/5*. As Nymphaeales is considered to have diverged from the other plants before the Eudicot-Monocot split (Zhang L et al., 2020), the *PRR5/9* duplication event in eudicots may have happened before the γ polyploidy event. However, the PRR5/9 genes in water lilies are more similar to the monocots genes by sequence identity and positional orthology (Figures 1 and 3), suggesting that this group’s history may be more complicated than expected. 

When analysing the *PRR9/5* genes in eudicots using positional orthology (Figure 4), it is possible to notice that a *LATE EMBRYOGENIS ABUNDANT PROTEIN 2* (*LEA2*) flanks most *PRR9/5*. A *bHLH57* transcription factor also flanks one group (Figure 4A), and a *30S RIBOSOMAL PROTEIN SUBUNIT* flanks the other (Figure 4B). In monocots, one group is flanked by *LEA2,* and a *PENTATRICOPEPTIDE REPEAT PROTEIN* (*PPR*) gene or a *PROTEIN STAY GREEN* (Figure 4C), while an *EXOCYTOSIS COMPONENT 70* (*EXO70*) gene flanks the other (Figure 4D). 

When analysing the *PRR7/3* in eudicots using positional orthology (Figure 3), most genes have a *PROTEIN KINASE* within 1 to 3 genes. In addition, the *PRR7/3* can be divided into two groups: a larger group that is also flanked by the genes for a *NUCLEOTIDE KINASE*, a *GLUTATHIONE S-TRANSFERASE* and/or TBC domain-containing protein (Figure 3A), and a smaller group that is also flanked by the genes for an *ALCOHOL DEHYDROGENASE*, a *HYDROLASE* and/or a *MAPK* (Figure 3B). The genes from the larger group can be found in all the eudicots and duplicated in *Populus trichocarpa* Torr. & A. Gray ex Hook*.* (Malpighiales) (*PtPRR37* and *PtPRR73*), *Solanum lycopersicum* L. (Solanales) (*SlPRR37* and *SlPRR37-like*) and *Beta vulgaris* L. (beets, Caryophyllales) (*BvPRR7* and *BvBTC1*). *S. lycopersicum* also has one gene that does not fit either group (*SlPRR73*). The genes from the smaller group are restricted to the Rosids, including *Carica papaya* L. (Brassicales) and *Vitis vinifera* L. (Vitales) (Figure 3B), and *Citrus clementina* Hort. ex Tan. (Sapindales), *Medicago truncatula* Gaertn. (Fabales) and *Theobroma cacao* L. (Malvales) (not shown). Non-rosid eudicots with two genes, such as beets, have duplications in the larger group (*BvPRR7* and *BvBTC1*) and none in the smaller group (Pin et al., 2012). AtPRR3 does not fit either group, even though it is usually associated with the smaller group. A *PROTEIN KINASE* also flanks *PRR7/3* genes in monocots. They can be divided into two groups of similar size: one usually called *PRR37*, which is flanked by a gene for *ASCORBATE PEROXIDASE* (Figure 3C), and one called *PRR73*, flanked by the genes for a TBC domain-containing protein and a Ribosomal protein (Figure 3D).

## PRRs in crops

Circadian rhythms affect plant productivity (Dodd et al., 2005); thus, it is not surprising that they may have a role in Agriculture (Steed et al., 2021; Hotta, 2021). Crop domestication frequently leads to the selection of mutants in the circadian oscillator due to their effects on photoperiodic responses, such as flowering (Bendix et al., 2015; McClung, 2021). In *Hordeum vulgare* L. (barley, Poales), a cultivar with reduced response to photoperiod allowed the use of this crop in northern parts of Europe. These changes were associated with a mutation in the *Photoperiod-H1* (*Ppd-H1*) locus. Cloning this locus showed that the *ppd-H1* mutation is a single nucleotide change in the CCT domain of a *PRR7/3*, *HvPRR37* (Turner et al., 2005). This mutation changes the flowering time on long days but has no apparent effect on the circadian oscillator (Campoli et al., 2012). *Ppd-H1* is collinear with the *Ppd-D1* allele in *Triticum aestivum* L. (wheat, Poales), a Green Revolution mutation that turns wheat into a photoperiod insensitive plant (Beales et al., 2007). Mutations in the *PRR37* orthologs selected by breeding can also be found in *Sorghum bicolor* (L.) Moench (sorghum, Poales) (Murphy et al., 2011) and *Oryza sativa* (rice, Poales) (Koo et al., 2013). 

Mutations in genes belonging to the PRR7/3 clade were also selected in eudicot crops. The domestication of beets selected a rare allele of *BvBTC1*, an ortholog from the PRR7/3 clade, that reduces the sensitivity to photoperiod (Pin et al., 2012). As this sensitivity reduction is reverted by vernalisation, beets with a mutated *Bvbtc1* allele turn from an annual to a biannual crop (Pin *et al.*, 2012). During the domestication of *Glycine max* (L.) Merr. (soybeans, Fabales), changes in a pair of *PRR7/3* orthologs (*GmPRR3A* and *GmPRR3B*) led to the loss of their CCT domain, resulting in the earlier flowering and reduction of the growth period (Li et al., 2019; Li and Lam, 2020).

## The role of PRRs in green plants

Apart from *TOC1/PRR1*, the role of the *PRRs* in the circadian oscillator is not fully understood. In Arabidopsis, the *PRR*s are considered part of the three interlocked loops of the core oscillator (Pokhilko et al., 2012). AtTOC1 is part of the core loop with AtLHY/AtCCA1 (Alabadí et al., 2001) and the evening loop with the EC (Pokhilko *et al.*, 2012). AtPRR7, AtPRR9 and AtPRR5 are part of the morning loop with LHY/CCA1 (Farré et al., 2005; Nakamichi et al., 2010) while also interacting with the EC (Chow et al., 2012; Pokhilko *et al.*, 2012). Mutation in *AtTOC11* or *AtPRR5* leads to a short period (Millar et al., 1995; Yamamoto et al., 2003), while a mutation in *AtPRR9* or *ATPRR7* leads to an extended period (Eriksson et al., 2003; Michael et al., 2003; Yamamoto *et al.*, 2003). Arrhythmia is only observed in the triple mutant *Atprr5 Atprr7 Atprr9* in constant conditions (Nakamichi *et al.*, 2005). The triple mutant also shows less photoperiodic and photomorphogenic responses (Nakamichi *et al.*, 2005). The PRRs act as transcriptional inhibitors by binding to the DNA through their CCT domains (Nakamichi *et al.*, 2010; Gendron et al., 2012; Nakamichi *et al.*, 2012). Thus, the waves of expression of *PRRs* regulate the transcription of genes throughout the day. For example, AtPRR5 targets are repressed from noon until midnight (Nakamichi *et al.*, 2012). However, in monocots, no changes in the circadian oscillator were observed when some genes from the PRR3/7 clade were mutated to change flowering, suggesting subfunctionalisation. For example, changes in *OsPRR73* did not lead to changes in flowering, nor did changes in *OsPRR37* lead to changes in the circadian oscillator (Murakami et al., 2003; Higgins et al., 2010). 

**Figure 5-:**
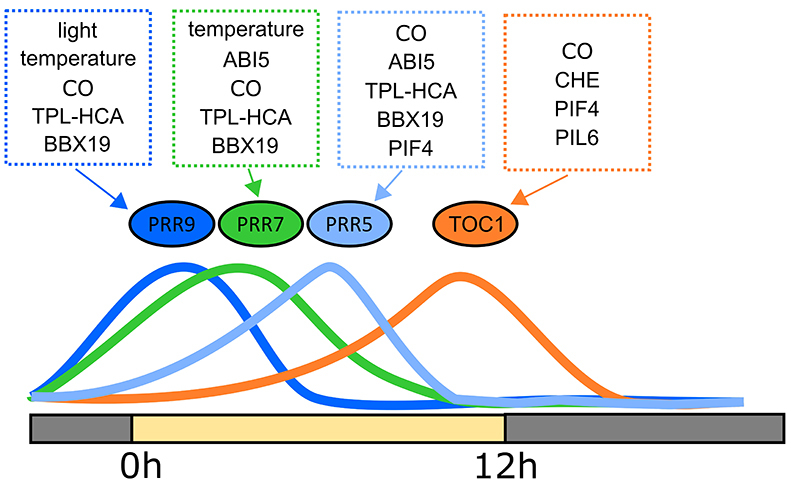
Regulators of the PRR proteins in *Arabidopsis thaliana*. AtPRR9 (dark blue), AtPRR7 (green), AtPRR5 (light blue) and AtTOC1 (orange) are expressed during the daytime, forming waves of expression. The PRR proteins make protein-protein complexes that regulate their DNA binding activity.

There is increasing evidence that PRRs act by forming protein complexes to regulate gene expression (Figure 5). In the core oscillator, during the night, AtTOC1 interacts with the TCP transcription factor AtCHE to inhibit *AtCCA1* expression by binding to its promoter (Pruneda-Paz et al., 2009). Other PRR-protein complexes also inhibit *AtCCA1* expression: at dawn, the Groucho/Tup1 corepressors TOPLESS (AtTPL) and TOPLESS-RELATED (AtTPR) form protein complexes with HISTONE DEACETYLASE 6 (AtHDA6), and AtPRR9, AtPRR7 or AtPRR5. The TPL-PRR-HDC complex bind inhibits *AtCCA1* and *AtLHY* expression by directly binding to their promoter (Wang et al., 2013). Later in the day, the B-box zinc-finger transcription factor AtBBX19 forms protein complexes with AtPRR9, AtPRR7 and AtPRR5 to regulate the period of the core oscillator, also by inhibiting *AtCCA1* expression (Yuan et al., 2021). The concerted action of the PRRs and their binding partners restrict *CCA1*/*LHY* expression to the first hours of the day. As CCA1/LHY regulates the expression of several Arabidopsis genes, PRR-protein complexes are essential to regulate the phase of transcriptional rhythms during the day. AtPRR9, AtPRR7 and AtPRR5 sequentially interact with PHYTOCHROME INTERACTING FACTORS (PIFs) to repress their induction of growth-related genes, such as the transcription factor *CYCLING DOF FACTOR 5* (*AtCDF5*). *AtCDF5* transcription is induced by PIFs before dawn, inducing cell elongation (Martín et al., 2018). In addition, AtTOC1 and AtPRR5 suppress thermomorphogenesis by interacting with AtPIF4 (Zhu et al., 2016). Thus, PRRs can be a gating mechanism that regulates plant growth. Gating is the regulatory mechanism that changes plant responses to signals due to the time of the day (Hotta et al., 2007). Shade-avoided responses are gated by PRRs, as AtPRR5 and AtPRR7 directly interact with other PIF proteins, and AtTOC1 directly interacts with PIF3-LIKE 1 (PIL1) (Salter et al., 2003; Zhang Y et al., 2020). Consequently, the maximum response is observed at dusk, when TOC1 levels are highest (Salter *et al.*, 2003).

The PRR-protein interactions also regulate flowering in Arabidopsis. The accumulation of AtCO at the end of the day triggers flowering by promoting *FLOWERING LOCUS T* (*AtFT*) expression (Valverde et al., 2004). The circadian oscillator regulates *AtCO* transcription, but protein levels of AtCO are independently stabilised by photoreceptors and PRRs (Valverde *et al.*, 2004; Hayama et al., 2017). The binding of the PRRs to AtCO also increases its binding to the At*FT* promoter (Hayama *et al.*, 2017). In monocots, PRR7/3 orthologs are associated with flowering initiation or repression (Turner et al., 2005; Beales et al., 2007; Murphy et al., 2011). In barley, HvCO1 activates *HvFT*, triggering flowering under long days (LD). This activation is made stronger by HvPRR37 (Ppd-H1), even though it does not regulate *HvCO1* transcription levels (Campoli et al., 2012). In contrast, SbPRR37 inhibits *SbCO* under LD in sorghum, a short-day plant (Yang et al., 2014). Similarly, OsPRR37 inhibits *OsFT* (*H3a*) expression under LD in rice (Koo et al., 2013).

Other outputs directly regulated by PRRs are the inhibition of photomorphogenic responses to red light, mediated by the interaction between AtTOC1 and AtPIL6 (Fujimori et al., 2004), and abscisic acid (ABA) signalling during germination, mediated by AtPRR5 and AtPRR7 and AtABI5 (Yang et al., 2021). ABA signalling also forms a feedback loop with AtTOC1 (Legnaioli et al., 2009; Lee et al., 2016).

The protein complexes formed by PRRs can also act as input pathways to the core oscillator, integrating information about light, temperature, and energy status. AtPRR9 is light-responsive but not the other PRRs, and thus it is one point of entry of light signalling into the core oscillator (Farré et al., 2005; Ito et al., 2005; Zeilinger et al., 2006). Double mutants of *AtPRR7* and *AtPRR9* cannot entrain to temperature changes, nor can they compensate for temperature, suggesting that these genes are part of the temperature input pathways into the circadian oscillator (Salomé and McClung, 2005; Salomé *et al.*, 2010). Finally, energy status regulates the circadian oscillator by inhibiting AtPRR7 through the transcription factor AtbZIP63 downstream of the SnRK1/KIN10 signalling pathway (Haydon et al., 2013; Frank et al., 2018; Viana et al., 2021).

## Conclusions

The PRR gene family is an integral part of the circadian oscillator, with a role in the core oscillator and the input and output pathways. The PRRs can make protein-protein and protein-DNA interactions, interacting with many proteins and promoters. The three clades of PRRs have a different evolutionary history, with only one copy of *TOC1/PRR1* in Angiosperms and multiple copies of *PRR7/3* and *PRR9/5*. When the numerous genome-wide duplications are considered, many copies of these genes were lost, probably to maintain the correct gene dosage. However, evidence of subfunctionalisation of the *PRR7/3* clade in monocots suggests that the roles of these genes may vary among the different plant species. Consequently, sequence similarities and mutant complementation using heterologous genes may not be enough to establish functional homology among other species. The function of these genes may not lie in their structure but in their protein and DNA binding partners. Until most of the protein complexes formed by PRRs are described, it will be difficult to fully understand the whole function of PRR proteins in the plant circadian clock.

## Supplementary material

The following online material is available for this article:

Table S1 - List of PRR orthologs used for sequence analysis.
