# Supplementary material for: The evolution and function of the PSEUDO RESPONSE REGULATOR gene family in the plant circadian clock
Source: Genet Mol Biol. 2022 Sep 16;45(3 Suppl 1):e20220137. doi: 10.1590/1678-4685-GMB-2022-0137 (PMC9486492; doi:10.1590/1678-4685-GMB-2022-0137)
Supplement: Table S1 - [file 1415-4757-GMB-45-3-s1-e20220137-s1.pdf]

Genetics and Molecular Biology

https://doi.org/10.1590/1678-4685-GMB-2022-0137

Supplementary Material to "The evolution and function of the *PSEUDO RESPONSE REGULATOR* gene family in the plant circadian clock"

Table S1 – List of PRR orthologs used for sequence analysis. Sequences can be found at Phytozome 3.0.

Gene name	Species	Exons	Gene ID
**OtTOC1**	*Ostreococcus tauri*	2	ostta13g01820
**MpTOC1**	*Marchantia polymorpha*	6	Mapoly0085s0081
**MpPRR**	*Marchantia polymorpha*	7	Mapoly0122s0007
**PpPRR1**	*Physcomitrium patens*	7	Pp3c25_4920
**PpPRR2**	*Physcomitrium patens*	9	Pp3c16_18460
**PpPRR3**	*Physcomitrium patens*	7	Pp3c25_5040
**PpPRR4**	*Physcomitrium patens*	9	Pp3c16_18610
**NcTOC1**	*Nymphaea colorata*	8	Nycol.H00282
**NcPRR7**	*Nymphaea colorata*	8	Nycol.E00505
**NcPRR9a**	*Nymphaea colorata*	8	Nycol.J00669
**NcPRR9b**	*Nymphaea colorata*	8	Nycol.A00921
**AtTOC1**	*Arabidopsis thaliana*	6	AT5G61380
**AtPRR3**	*Arabidopsis thaliana*	9	AT5G60100
**AtPRR5**	*Arabidopsis thaliana*	6	AT5G24470
**AtPRR7**	*Arabidopsis thaliana*	8	AT5G02810
**AtPRR9**	*Arabidopsis thaliana*	7	AT2G46790
**CpTOC1**	*Carica papaya*	2	evm.TU.supercontig_13.294
**CpPRR5a**	*Carica papaya*	8	evm.TU.supercontig_3.152
**CpPRR5b**	*Carica papaya*	10	evm.TU.supercontig_193.20
**CpPRR7a**	*Carica papaya*	8	evm.TU.supercontig_1.291
**CpPRR7b**	*Carica papaya*	10	evm.TU.supercontig_139.32
**PtTOC1**	*Populus trichocarpa*	6	Potri.015G061900
**PtPRR37**	*Populus trichocarpa*	8	Potri.008G046200
**PtPRR73**	*Populus trichocarpa*	8	Potri.010G215200
**PtPRR5a**	*Populus trichocarpa*	8	Potri.012G005900
**PtPRR5b**	*Populus trichocarpa*	8	Potri.015G002300
**PtPRR9la**	*Populus trichocarpa*	8	Potri.002G179800
**PtPRR9lb**	*Populus trichocarpa*	8	Potri.014G106000
**VvTOC1**	*Vitis vinifera*	6	VIT_217s0000g06570
**VvTOC1a-like**	*Vitis vinifera*	3	VIT_217s0000g06560
**VvTOC1b-like**	*Vitis vinifera*	4	VIT_217s0000g06520
**VvPPR7_1**	*Vitis vinifera*	10	VIT_206s0004g03650
**VvPRR7_2**	*Vitis vinifera*	8	VIT_213s0067g03390

Gene name	Species	Exons	Gene ID
**VvPPR9**	*Vitis vinifera*	8	VIT_215s0048g02540
**VvPRR5**	*Vitis vinifera*	8	VIT_216s0098g00900
**SlTOC1**	*Solanum lycopersicum*	6	Solyc03g115770
**SlPRR37**	*Solanum lycopersicum*	7	Solyc04g049670
**SlPRR37-like**	*Solanum lycopersicum*	2	Solyc04g049680
**SlPRR73**	*Solanum lycopersicum*	8	Solyc10g086000
**SlPRR95**	*Solanum lycopersicum*	8	Solyc10g005030
**SlPRR59**	*Solanum lycopersicum*	8	Solyc03g081240
**BvTOC1**	*Beta vulgaris*	4	EL10Ac5g11059
**BvPRR7**	*Beta vulgaris*	10	EL10Ac7g17317
**ScBTC1**	*Beta vulgaris*	8	EL10Ac2g03535
**BvPRR5a**	*Beta vulgaris*	8	EL10Ac1g00184
**BvPRR5b**	*Beta vulgaris*	8	EL10Ac3g05174
**OsTOC1**	*Oryza sativa*	6	LOC_Os02g40510
**OsPRR37**	*Oryza sativa*	8	LOC_Os07g49460
**OsPRR73**	*Oryza sativa*	8	LOC_Os03g17570
**OsPRR95**	*Oryza sativa*	8	LOC_Os09g36220
**OsPRR59**	*Oryza sativa*	8	LOC_Os11g05930
**BdTOC1**	*Brachypodium distachyon*	6	Sobic.004G216700
**BdPRR37**	*Brachypodium distachyon*	8	Sobic.006G057866
**BdPRR73**	*Brachypodium distachyon*	8	Sobic.001G411400
**BdPRR95**	*Brachypodium distachyon*	7	Sobic.002G275100
**BdPRR59**	*Brachypodium distachyon*	8	Sobic.005G044400
**SbTOC1**	*Sorghum bicolor*	6	Sevir.1G241000
**SbPRR37**	*Sorghum bicolor*	5	Sevir.2G456400
**SbPRR73**	*Sorghum bicolor*	8	Sevir.9G449300
**SbPRR5a**	*Sorghum bicolor*	8	Sevir.2G296500
**SbPRR5b**	*Sorghum bicolor*	8	Sevir.8G038500
**SvTOC1**	*Setaria viridis*	6	Bradi3g48880
**SvPRR37**	*Setaria viridis*	8	Bradi1g16490
**SvPRR73**	*Setaria viridis*	8	Bradi1g65910
**SvPRR95**	*Setaria viridis*	8	Bradi4g36077
**SvPRR59**	*Setaria viridis*	8	Bradi4g24967
